# DVA: predicting the functional impact of single nucleotide missense variants

**DOI:** 10.1186/s12859-024-05709-6

**Published:** 2024-03-06

**Authors:** Dong Wang, Jie Li, Edwin Wang, Yadong Wang

**Affiliations:** 1 School of Computer Science and Technology, Harbin Institute of Technology Harbin, Harbin, Heilongjiang China; 2 Cumming School of Medicine, University of Calgary, Calgary, Canada

**Keywords:** Missense variants, Functional impact, Variant annotation, Disease-related

## Abstract

**Background:**

In the past decade, single nucleotide variants (SNVs) have been identified as having a significant relationship with the development and treatment of diseases. Among them, prioritizing missense variants for further functional impact investigation is an essential challenge in the study of common disease and cancer. Although several computational methods have been developed to predict the functional impacts of variants, the predictive ability of these methods is still insufficient in the Mendelian and cancer missense variants.

**Results:**

We present a novel prediction method called the disease-related variant annotation (DVA) method that predicts the effect of missense variants based on a comprehensive feature set of variants, notably, the allele frequency and protein–protein interaction network feature based on graph embedding. Benchmarked against datasets of single nucleotide missense variants, the DVA method outperforms the state-of-the-art methods by up to 0.473 in the area under receiver operating characteristic curve. The results demonstrate that the proposed method can accurately predict the functional impact of single nucleotide missense variants and substantially outperforms existing methods.

**Conclusions:**

DVA is an effective framework for identifying the functional impact of disease missense variants based on a comprehensive feature set. Based on different datasets, DVA shows its generalization ability and robustness, and it also provides innovative ideas for the study of the functional mechanism and impact of SNVs.

## Background

With the rapid accumulation of the Human Genome Sequencing Project [1–5], more than millions of human genetic variations have been discovered and stored by researchers. Among them, the prediction of functional impact of missense variants is interesting and critical research field. The meaning of functional impact is that a variant that may increase an individual’s susceptibility or predisposition to a certain disease or disorder. For example, SNVs located in the coding region of the DNA sequence may lead to the different amino acid translation, resulting in the impact on the function of gene products in multiple ways, e.g., by affecting the protein’s interaction with other proteins, its stability or it active sites. These changes may have an important impact on the overall function and signal transmission of the gene product. Therefore, how to accurately interpret the functional impact of missense variants is particularly important.

Various computational methods have been proposed to predict the functional impact of variants. Prediction methods based on a single type of information (for example, conservation/evolution/sequence homology) were proposed earlier. For example, Kumar et al. [6] proposed a prediction method called SIFT that predicts the effects of missense variants (amino acid substitutions, AASs) based on the sequence homology and the main idea of SIFT is that highly conservative positions tend to not tolerate variants/substitutions, while low conservative positions are just opposite. The prediction method based on single information can provide a simple, intuitive and easy to explain prediction result, which is convenient for relevant researchers and clinical staff to use in practical work. However, its prediction results are often less accurate. Therefore, many methods have been developed to improve prediction accuracy by merging multiple types of variant features. For example, Shihab et al. [7] developed a novel method called FATHMM-MKL that integrated 10 different variant features (such as sequence conservation, histone modification, footprints, GC content, transcription factor binding sites) to improve the accuracy of functional impact prediction of variants. In addition to combining the features of variants, some methods also take the prediction scores of other methods as features to improve prediction accuracy. Such as REVEL [8] and MISTIC [9], these methods all use the scores of other prediction methods as features to predict the functional impact of variants. These different types of computational methods have advanced the study of the prediction of the functional impact of variants. However, most of these methods do not perform well enough [10] in the functional impact prediction of missense variants. Therefore, how to construct a comprehensive feature set and an efficient computational model to improve the prediction accuracy is still a key challenge.

To solve the above problems, we proposed a method called disease-related variant annotation (DVA, http://bionet.org.cn/DVA), which systematically integrated multiple features including conserved elements, allele frequencies in different populations, and protein–protein interaction (PPI) network feature transformation. The sequence conservation (DNA or protein) often means that a sequence has been maintained by natural selection and is considered to have functional value [11–13]. Therefore, when a variant or its corresponding amino acid substitution occurs at a highly conserved site, the possibility of harmful effects often is greater than the possibility of harmlessness. In addition to sequence conservation, allele frequency is also used as a predictor, that is, low-frequency variants usually trend to be disease-related, and high-frequency variants trend to be neutral [14]. Here, allele frequency is used as a predictor variable to predict the functional impact of variants. Except for the perspective of single-site and population statistics, the important impact of missense variants may lie in the interaction of protein molecules. For a protein molecule to perform a certain function, it often needs to be combined or coordinated with other protein molecules. However, previous methods rarely take this into account. Therefore, we employ this (PPI network) as the novel feature to predict the functional impact of variants, which is extracted by graph embedding. To sum up, the DVA method constructs a comprehensive feature set including sequence conservation, allele frequency, and PPI structure feature to predict the functional impact of variants accurately. The novelty of our approach lies in the combination of a set of features including conserved elements, allele frequencies in different populations, and PPI network feature transformation, to build the random forest model that achieves the significant improvement of accuracy within different complex diseases and cancers using missense variant datasets.

## Results and discussion

### Experimental results for somatic cancer missense variants

**Fig. 1:**
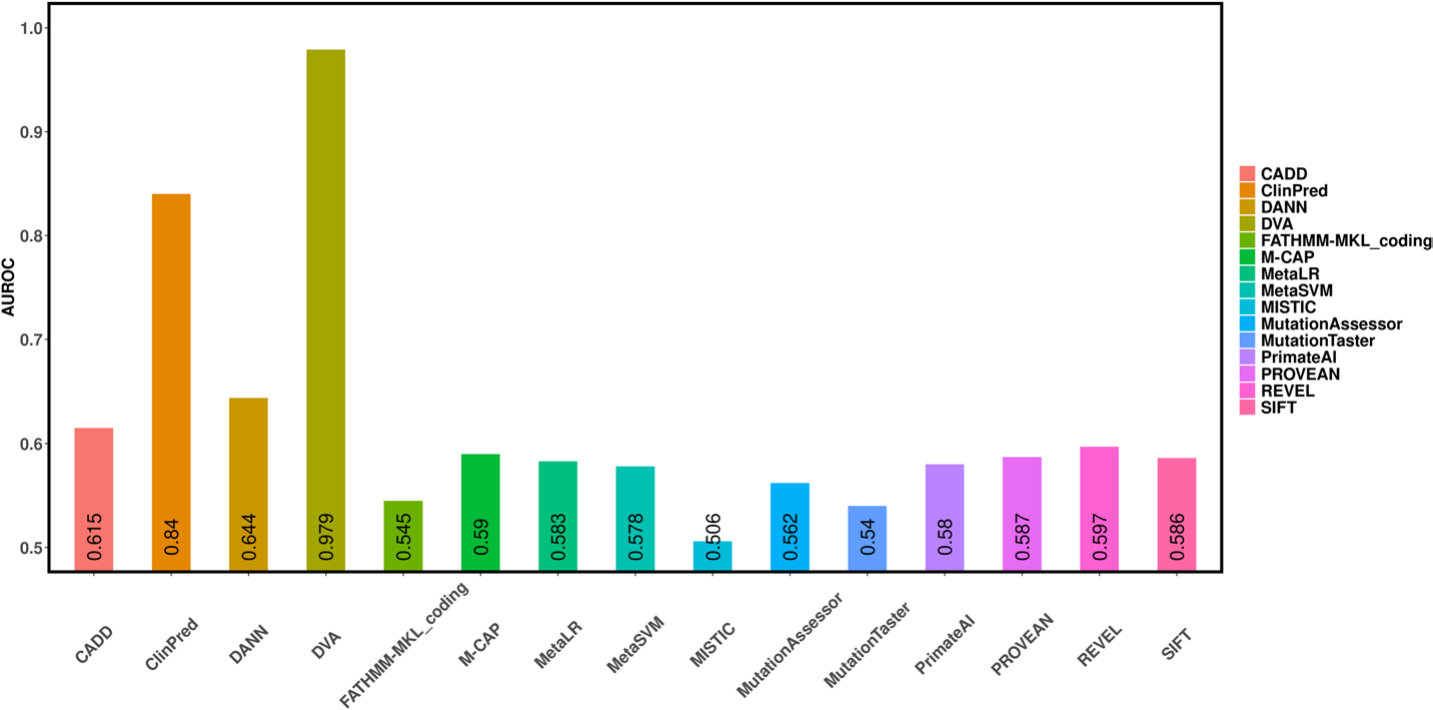
The AUROCs of 15 different prediction methods using somatic cancer missense variants

Experimental results on somatic cancer variants are shown in Fig. 1 and Table 1. The area under receiver operating characteristic curve (AUROC) for DVA is 0.979. Here, we compared the DVA method to 14 prediction methods that were recently developed, widely used: SIFT [6], PROVEAN [15], MutationTester [16], MutationAssessor [17], FATHMM-MKL [7], DANN [18], MetaSVM [19], MetaLR [19], ClinPred [14], CADD [20], PrimateAI [21], REVEL [8], M-CAP [22], and MISTIC [9]. The prediction scores of these methods were obtained from the webserver or software provided by authors, ANNOVAR, or the dbNSFP v3/v4 database. The AUROCs for the other individual prediction methods ranged from 0.506 to 0.84. Among them, the highest AUROC value was 0.84 achieved by the ClinPred method and the lowest AUROC value was 0.506 achieved by the MISTIC method. As a result, many prediction methods performed poorly on somatic cancer variants. Nevertheless, DVA significantly outperformed other functional impact prediction methods on such data. These results demonstrate that DVA has a good ability to predict the functional impact of somatic cancer variants.

**Table 1:** Performance of prediction methods using the somatic cancer missense variants

Order	Methods	Accuracy	Precision	Recall	F1-score
1	SIFT	0.587	0.525	0.435	0.468
2	MutationTaster	0.509	0.454	0.743	0.563
3	MutationAssessor	0.575	0.505	0.398	0.441
4	FATHMM-MKL_coding	0.564	0.49	0.442	0.46
5	PROVEAN	0.584	0.514	0.544	0.526
6	MetaSVM	0.579	0.511	0.481	0.49
7	MetaLR	0.57	0.498	0.569	0.529
8	DANN	0.646	0.6	0.517	0.555
9	CADD	0.62	0.578	0.437	0.491
10	MISTIC	0.585	0.539	0.248	0.333
11	REVEL	0.595	0.53	0.484	0.504
12	PrimateAI	0.604	0.553	0.405	0.463
13	ClinPred	0.795	0.811	0.679	0.739
14	M-CAP	0.581	0.512	0.535	0.52
15	**DVA**	**0.94**	**0.918**	**0.943**	**0.93**

The best results are bolded

In addition to the AUROC, accuracy, precision, recall, and F1-score also were employed to evaluate the performance of DVA and state-of-the-art prediction methods. As shown in Table 1, the performance of the previous algorithms was relatively poor and the DVA method has achieved the best performance among the four evaluation criteria (accuracy, precision, recall, and F1-score). In particular, the recall for DVA was higher than other methods by at least 0.2. The proposed method significantly outperformed other prognostic predictors of functional impact of somatic cancer variants.

### Experimental results for missense variants in ClinVar database

**Fig. 2:**
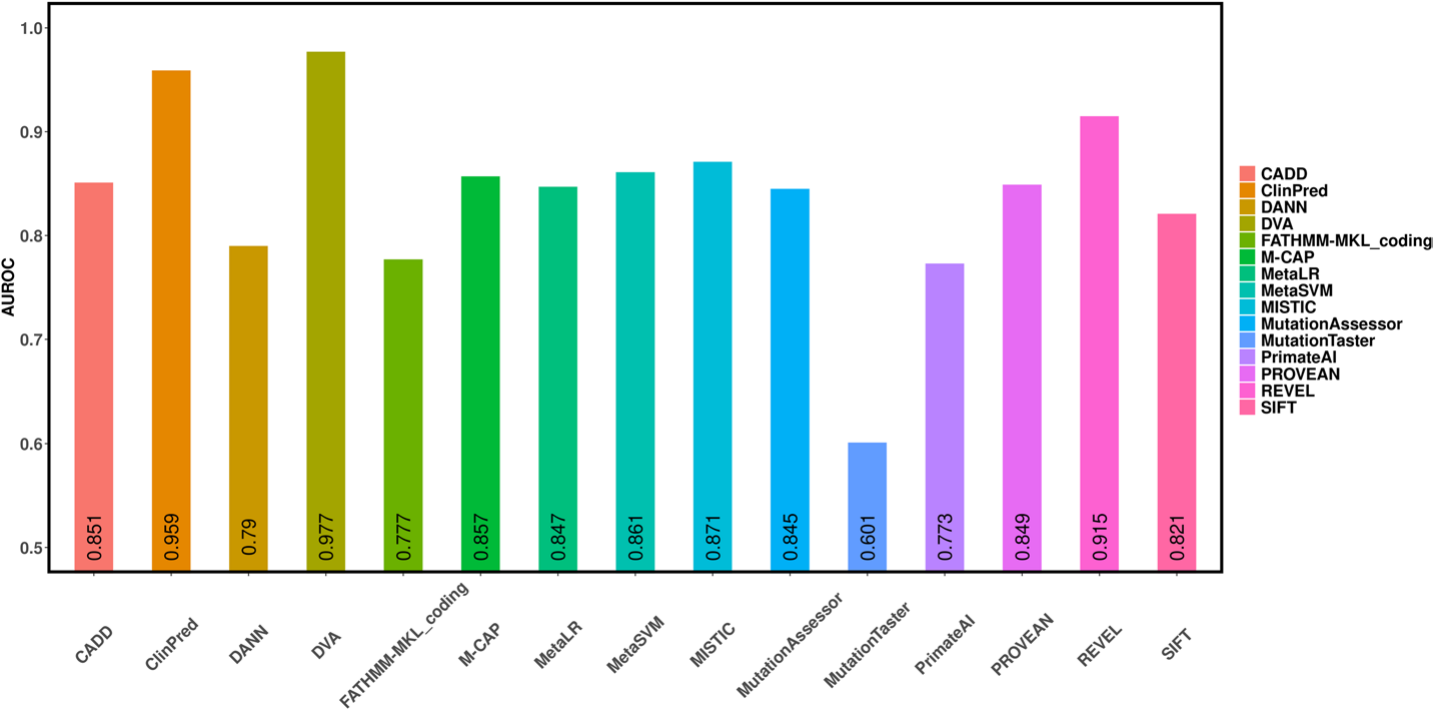
The AUROCs of 15 different prediction methods using missense variants in the ClinVar database

Experimental results on the ClinVar missense variants are shown in Fig. 2 and Table 2. The AUROC value of DVA is 0.977. The AUROC for the other individual prediction methods ranged from 0.601 to 0.959. For example, REVEL and ClinPred achieve relatively good performance, with AUROC values of 0.915 and 0.959, respectively. The AUROCs of other prediction tools fluctuate around 0.8 (AUROC for MetaSVM is 0.861; AUROC for MetaLR is 0847; AUROC for CADD is 0.851; AUROC for DANN is 0.79; AUROC for PrimateAI is 0.773; AUROC for FATHMM-MKL is 0.777; AUROC for MutationAssessor is 0.845; AUROC for MISTIC is 0.871; AUROC for PROVEAN is 0.849; AUROC for SIFT is 0.821; AUROC for M-CAP is 0.857). MutationTaster has the worst predictive performance in the ClinVar missense variants with an AUROC of 0.601. These results demonstrate that DVA has a good ability to predict the functional impact of missense variants of different mendelian diseases.

**Table 2:** Performance of prediction methods using missense variants in the ClinVar database

Order	Methods	Accuracy	Precision	Recall	F1-score
1	SIFT	0.756	0.803	0.748	0.773
2	MutationTaster	0.629	0.624	0.846	0.718
3	MutationAssessor	0.776	0.836	0.747	0.788
4	FATHMM-MKL_coding	0.735	0.743	0.807	0.772
5	PROVEAN	0.794	0.818	0.814	0.815
6	MetaSVM	0.799	0.845	0.785	0.813
7	MetaLR	0.774	0.806	0.79	0.795
8	DANN	0.741	0.75	0.809	0.776
9	CADD	0.798	0.806	0.843	0.824
10	MISTIC	0.805	0.854	0.788	0.819
11	REVEL	0.85	0.889	0.837	0.862
12	PrimateAI	0.731	0.751	0.781	0.764
13	ClinPred	0.909	0.942	0.892	0.916
14	M-CAP	0.795	0.826	0.804	0.814
15	**DVA**	**0.929**	**0.946**	**0.926**	**0.935**

The best results are bolded

As shown in Table 2, the DVA method also has achieved the best performance compared to other prediction tools. The recall of DVA is 0.926 at least 3.4 percentage points higher than other prediction tools. The precision, accuracy, and F1-score of DVA also have achieved the excellent performance, which indicates that DVA is effective in representing and distinguishing the differences between disease-related and neutral variants.

### Experimental results for missense variants in VariBench database

**Fig. 3:**
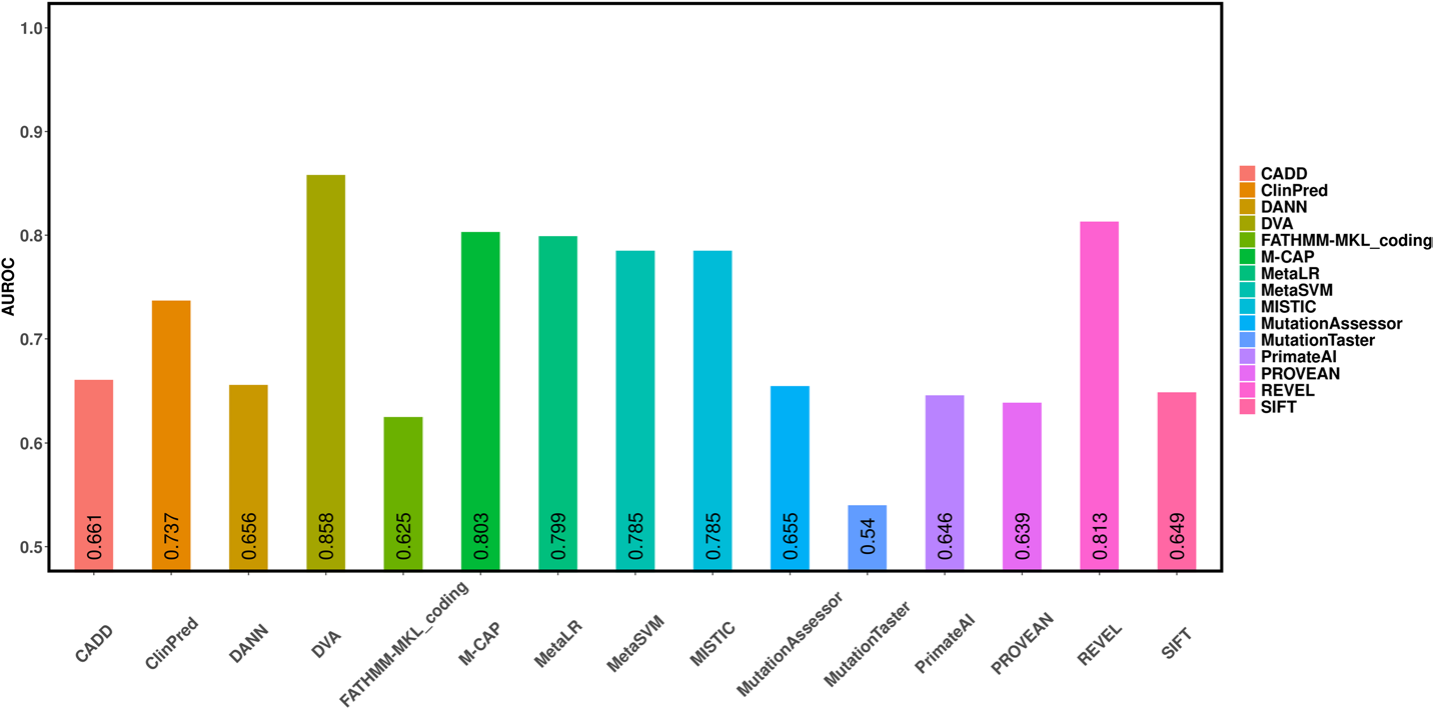
The AUROCs of 15 different prediction methods using missense variants in the VariBench database

Experimental results on the VariBench missense variants are shown in Fig. 3 and Table 3. As shown in Fig. 3, the DVA method has the best performance and its AUROC value is 0.858. For other prediction methods, the highest AUROC value was 0.813 achieved by the REVEL method and the lowest AUROC value was 0.54 achieved by the MutationTaster method. The deep neural network (DNN) has achieved an overwhelming advantage in some research fields of computer science, such as computer vision and natural language process. In the aspect of functional impact of variants, some prediction methods also employed deep neural networks. However, the performance of these methods did not significantly outperform other methods. DNN models require a lot of training data sets. Among all kinds of biological data, sequence data has a large scale to meet the requirements of training. Thus, PrimateAI employed a DNN model to predict the functional impact of variants using multi-sequences. Although DNN is used, the prediction result of PrimateAI based on sequence data is not excellent. DANN also is a prediction method based on the DNN model. Compared with other methods, DANN doesn’t show the significantly overwhelming performance too. Based on the current observations, DNN has not achieved significant success in this field. Thus, more excellent machine learning algorithms and feature sets may still be a better choice. These results demonstrate that DVA has a good ability to predict the functional impact of missense variants in VariBench database.

**Table 3:** Performance of prediction methods using missense variants in the VariBench database

Order	Methods	Accuracy	Precision	Recall	F1-score
1	SIFT	0.625	0.631	0.547	0.582
2	MutationTaster	0.536	0.512	**0.77**	0.615
3	MutationAssessor	0.631	0.65	0.523	0.573
4	FATHMM-MKL_coding	0.603	0.574	0.694	0.626
5	PROVEAN	0.617	0.611	0.594	0.593
6	MetaSVM	0.734	0.738	0.703	0.717
7	MetaLR	0.742	0.753	0.699	0.722
8	DANN	0.628	0.627	0.584	0.598
9	CADD	0.633	0.616	0.632	0.623
10	MISTIC	0.739	0.75	0.696	0.718
11	REVEL	0.755	0.751	0.741	0.744
12	PrimateAI	0.621	0.602	0.645	0.62
13	ClinPred	0.692	0.691	0.656	0.67
14	M-CAP	0.746	0.764	0.688	0.722
15	**DVA**	**0.785**	**0.797**	0.744	**0.768**

The best results are bolded

As shown in Table 3, the DVA method also has achieved the best performance compared to other prediction tools. The accuracy, precision, and F1-score of DVA also have the best performance compared to state-of-the-art methods. The prediction ability of the DVA method is also proved on the VariBench dataset.

### Experimental results for missense variants based on cross-database validation

**Fig. 4:**
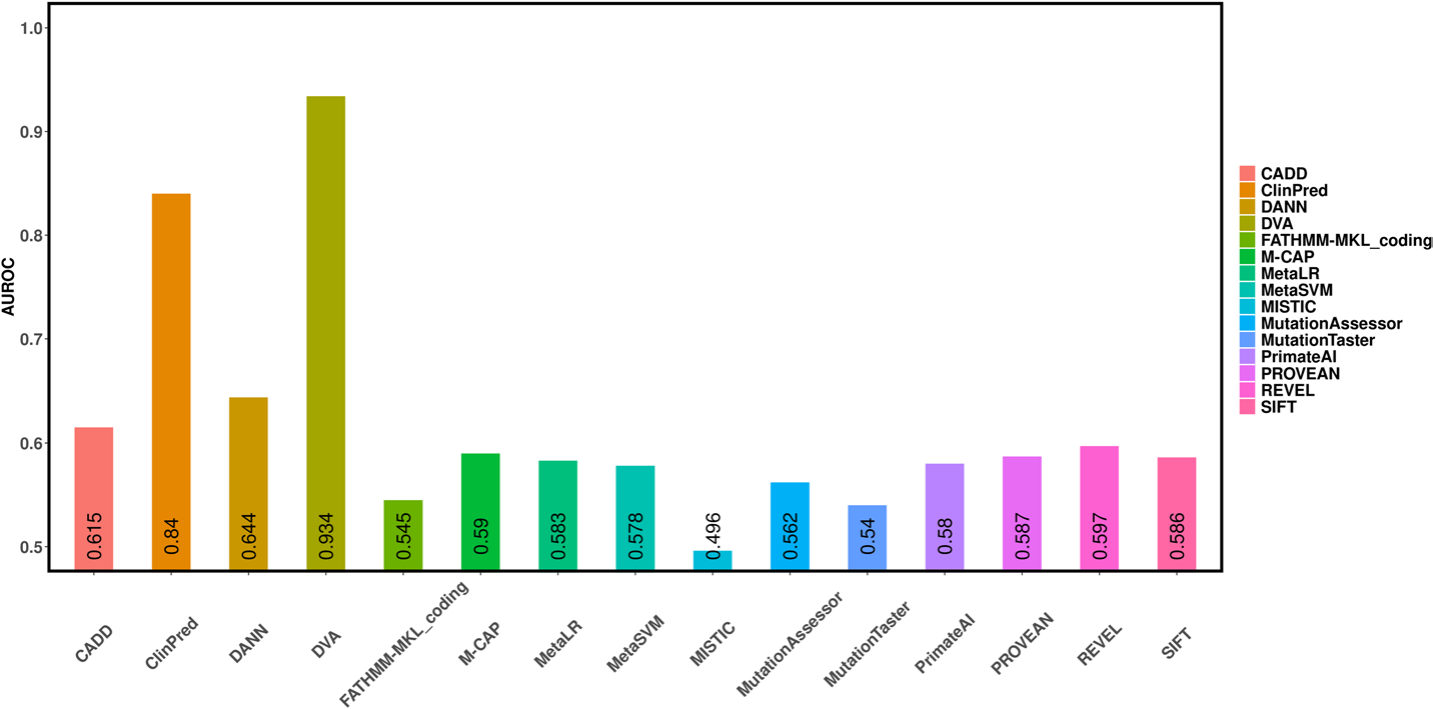
The AUROCs of 15 different prediction methods on COSMIC dataset

Prediction methods usually achieve better performance based the training data and testing data, which come from the same database. As shown in the Fig. 2 and Table 2, DVA achieves excellent performance (AUROC is 0.977) and the compared prediction methods also achieve good or fair performance (the AUROCs of most methods are around 0.85) based the training data and testing data, which come from the same database. However, when prediction methods are training in one dataset and testing in another dataset, the testing result may decrease. Thus, we perform another experiment to discuss whether DVA and other methods still have the better prediction ability when training data and test data come from different databases according to your suggestion. Most prediction methods (such as MISTIC and ClinPred) are trained on ClinVar or similar types of datasets. Thus, ClinVar and COSMIC/VariSNP were chosen as the training set and testing data, respectively. Experimental results are shown in the Fig. 4 and Table 4, the AUROC and accuracy of 15 methods have decreased. However, performance of other 14 methods is significantly reduced (the AUROCs of most methods are around 0.6), while DVA still achieves excellent performance (AUROC is 0.934). These results demonstrate that DVA has better robustness.

**Table 4:** Performance of prediction methods on COSMIC dataset

Order	Methods	Accuracy	Precision	Recall	F1-score
1	SIFT	0.568	0.495	0.537	0.515
2	MutationTaster	0.511	0.454	0.715	0.555
3	MutationAssessor	0.56	0.484	0.459	0.471
4	FATHMM-MKL_coding	0.567	0.491	0.397	0.439
5	PROVEAN	0.583	0.511	0.522	0.517
6	MetaSVM	0.577	0.505	0.473	0.488
7	MetaLR	0.571	0.498	0.527	0.512
8	DANN	0.647	0.603	0.507	0.551
9	CADD	0.617	0.568	0.428	0.488
10	MISTIC	0.569	0.492	0.293	0.367
11	REVEL	0.593	0.526	0.469	0.496
12	PrimateAI	0.603	0.551	0.372	0.444
13	ClinPred	0.793	0.803	0.682	0.737
14	M-CAP	0.58	0.507	0.521	0.514
15	**DVA**	**0.861**	**0.815**	**0.873**	**0.843**

The best results are bolded

### Feature importance analysis

**Fig. 5:**
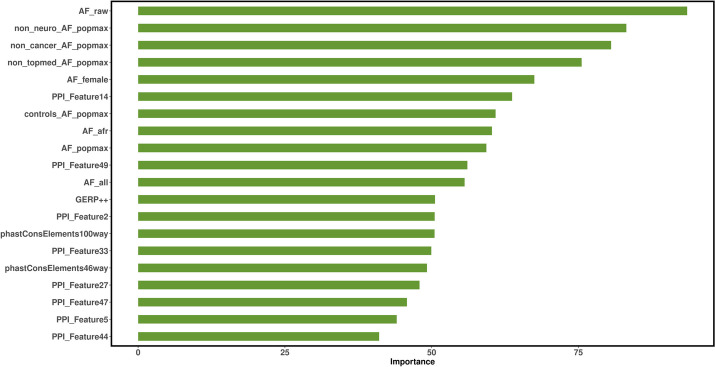
The degree of contribution from top 20 features used by DVA

We analyzed the importance of selected features for the prediction performance of DVA method and used *randomForest* package to obtain importance scores using missense variants in the VariBench dataset. Figure 5 represents the importance level of the top 20 features. Among the top 20 features, allele frequency features are the most important features in our method and accounting for 9 of the top 20 features. The PPI graph embedding features are next important features and accounting for 8 of the top 20 features. The importance of conservative score features is relatively lower than that of the first two types of features, but there are still three conservative features in the top 20 features. So, it has also played a certain role in predicting the functional impact of missense variants. From the above feature importance analysis, it can be concluded that the two new features performed in the DVA method play a significant role in predicting the functional impact of missense variants.

## Conclusions

Since the completion of human gene mapping, human beings have entered the post genome era. Annotation and analysis of gene and gene product functions are critical study at the post genome era. In addition to the general gene function research, the functional impact of variants occurred in the gene coding region is a very important and meaningful direction. For example, research by Shajani-Yi et al. [23] showed that some “key” genes have been found to carry disease-related variants in different cancers. In the Glioma/Glioblastoma cancer, 67% (16/24) of tumors exhibited one disease-related variant, of which 94% (15/16) were in IDH1 and 6% (1/16) were in PIK3CA. In the Colon Adenocarcinoma, 67% (73/109) of tumors with disease-related variants had more than one variant in addition to TP53. Daboub et al. [24] claimed a report of Parkes Weber syndrome associated with two disease-related variants in RASA1. Timms et al. [25] discovered that BRCA disease-related variants were observed in all breast cancer subtypes. These studies also show that disease-related variants have a greater correlation with cancer. However, there are several variants stored in different databases, which are not yet clear about their possible effects. Therefore, how to better predict disease-related variants can better enable researchers or clinicians to focus on the scope of cancer research, thereby promoting precision medicine. With DVA, we describe an effective framework for identifying the functional impact of disease missense variants based on a comprehensive feature set. We demonstrate that the performance of DVA is much better than the state-of-the-art prediction methods based on different missense variants. Meanwhile, we observed the general robustness of the DVA, and it will be innovative for the study of the functional mechanism and impact of SNVs.

New features and appropriate model may contribute to the improved prediction performance. First, two new types of features have been introduced to significantly improve the predictive ability of this algorithm: (a) variant allele frequencies in different populations. When a SNV is widely present in the population, it often has no pathogenic effect on the molecular function or organism. On the contrary, if a SNV only appears in a few individuals, its impact on the organism may be more pathogenic. It has been less considered in previous prediction methods. (b) PPI network features. Whether it is normal organic operations or harmful molecular changes, it is often not a single factor that promotes its development, but the interaction of multiple molecules or changes in key molecules that lead to essential changes in the entire working mechanism. In the past, little attention has been paid to the interaction of different molecules in the research of the functional impact of variants. Here we used the graph embedding (PPI network) to represent the interaction of molecules with different variants. Second, a random forest model was constructed to predict the functional impact of variants. By merging the different types of features and the random forest model, the DVA algorithm has significantly improved the prediction of the impact of variants.

In this article, the graph embedding representation of protein–protein interactions is used as a novel feature to predict functional impact of variants and it improves the performance of the proposed method effectively. In addition to protein–protein interaction, protein structure also is an informative and detailed feature to protein function and may improve the performance of prediction methods. However, applying protein structure to predict functional impact of variants is still challenging. There are several problems to be fixed: (1) Although the wild protein structures are accessible in several database [26–28], the mutated protein structure usually is not provided; (2) Protein structure is complicated. How to construct protein structure representation as the input feature of machine learning methods is still a critical challenge. In the future, we will continue to develop novel methods which focus on addressing existing problems to predict functional impact of variants.

## Methods

### DVA datasets

In this study, we used three datasets (ClinVar(v20210922) [3], VariBench [29] and COSMIC [4]/VariSNP(v2017) [30]) to assess the performance of the proposed method and the state-of-the-art prediction methods. All of datasets were categorized as the disease-related and neutral missense variants, which were selected as the positive (disease-related or recurrent in cancer tissues) and negative (neutral) labels, respectively. To evaluate the performance of DVA and the state-of-the-art prediction methods appropriately, we established the following rules to filter out genetic variants:

1. The functional impact of variants contained in benchmark datasets should be predictable by all the state-of-the-art prediction methods.
2. The variants should be rare, namely, the gnomAD allele frequencies (AF) of these variants are less than 1%.
3. Each variant should not occur in the training set of the state-of-the-art prediction methods.

Finally, we obtained three datasets: (1) 12,569 recurrent missense variants from the COSMIC (https://cancer.sanger.ac.uk/cosmic) dataset and 16,873 neutral missense variants from the VariSNP database; (2) 3706 disease-related missense variants and 2929 neutral missense variants from the ClinVar database; (3) 2965 disease-related missense variants and 3198 neutral missense variants from the VariBench database, which is integrated into the filtered versions of HumVar [31], ExoVar [32], VariBench, and SwissVar [33] datasets. There are some overlaps between the original databases. Through data preprocessing, we give priority to retaining duplicate variant data in ClinVar, then retaining variant data in Varibench, and finally retaining variant data in COSMIC and VariSNP.

### Performance evaluation

The performance of the state-of-the-art prediction methods and DVA was evaluated using accuracy, precision, recall, F1-score, and the area under the ROC curve:

1 $$Recall = \frac{TP}{{TP + FN}}$$

2 $$Precision = \frac{TP}{{TP + FP}}$$

3 $$F1 - score = \frac{2*Recall*Precision}{{Recall + Precision}}$$

4 $$Accuracy = { }\frac{TP + TN}{{TP + FP + TN + FN}}$$

In the equations above, the following parameters are defined: True Positive (TP), True Negative (TN), False Positive (FP), and False Negative (FN). The positive cases denote the disease-related, deleterious, or pathogenic missense variants, while the negative cases denote the neutral or benign missense variants. Accuracy is the rate at which the prediction method correctly classifies the positive and negative cases. The Precision and Recall represent the ratio of real positive cases to predicted positive cases and correctly predicted positive to correctly predicted cases, respectively. F1-score is a compromise between precision and recall. The Receiver Operating Characteristic (ROC) curve is a plot that illustrates the predictive ability of the prediction method. The Area Under the ROC curve (AUROC) is a numerical representation of the ROC curve to indicate the performance of the prediction method more conveniently. The AUROC, accuracy, precision, recall, and F1-score were obtained using the pROC [34] package implemented by the R language.

### Feature matrix construction

**Fig. 6:**
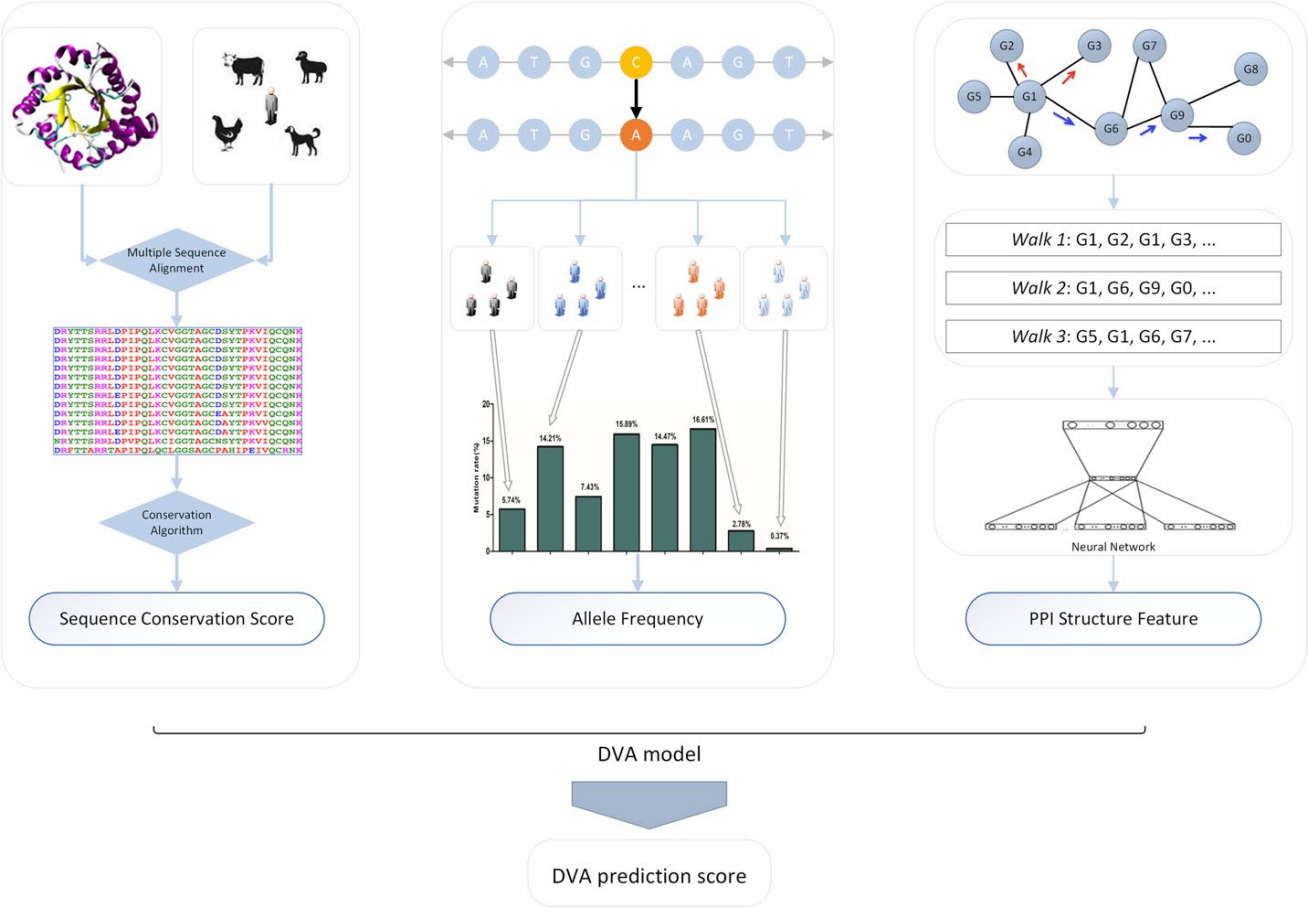
The overview of the DVA method

In this section, we will introduce the comprehensive feature set of DVA. There are three kinds of variant features: conserved element features, allele frequency features, and PPI network features as shown in the Fig. 6.

#### Conserved element features

Here, the DVA algorithm incorporates a total of 8 conserved element features as predictive features to identify the functional impact of missense variants: The Genomic Evolutionary Rate Profiling++ (GERP++) [35] discoveries constrained elements in multiple alignments by quantifying substitution deficits, which represent substitutions that would have occurred if the element were neutral but did not occur because the element has been under functional constraint. GERP++ is widely used as the common feature in the prediction of functional impact of variants. In addition to GERP++, The PHylogenetic Analysis with Space/Time models (PHAST) [36] and phyloP [37] and transcription conservation element are also integrated into DVA features, which increases the diversity of feature sets. All these conserved element features were obtained from the ANNOVAR database.

#### Allele frequency features

Complex diseases may be associated with low-frequency genetic variants [38]. Thus, we incorporated the allele frequency data including 17 features from the Genome Aggregation Database [39] (gnomAD version v2.1.1), such as AF_male, AF_female, AF (all), AF_afr (African/African-American), AF_sas (South Asian), AF_amr (Latino/Admixed American), AF_eas (East Asian), AF_nfe (Non-Finnish European), AF_fin (Finnish), AF_asj (Ashkenazi Jewish), AF_oth (Other) and so on. If the AF (all) of an SNV is missing, we will remove the SNV in subsequent experiments. Here, we fill in the missing values of conserved elements and AFs using filling [40] R package. All of the features were obtained using ANNOVAR [41] and dbNSFP v3/v4 [42, 43].

#### PPI network features

When a variant occurs in the “multi-connected” gene that has more neighbor genes in the PPI network, its impact may be greater. Therefore, we introduce the characteristics of the PPI network into the prediction of SNV function effects to improve accuracy. However, the dimension of PPI network is too high, which will undoubtedly increase the computational complexity, it will affect the prediction work. The graph embedding [44] based on network structure and random work is a good way to solve this problem. The specific steps are as follows:

- Based on protein–protein interaction database (STRING [45]), the binary adjacency matrix of PPI network will be obtained.
- A special random walk with two parameters $p$ and $q$ is performed to guide the walker. The transition probability is as follows:
  5 $$\beta_{pq} \left( {t,v,x} \right) = \left\{ {\begin{array}{*{20}c} \frac{1}{p} & {d_{tx} = 0} \\ 1 & {d_{tx} = 1} \\ \frac{1}{q} & {d_{tx} = 2} \\ \end{array} } \right.$$

where $\beta_{pq} \left( {t,v,x} \right)$ is the transition probability between current node $v$ and its neighbor node $x$. The node $t$ is the last step node, which is passed by the random walker. The $p$ and $q$ are walk direction parameter, respectively. Parameter $d_{tx}$ is the distance of node $t$ and node $x$. The $\beta_{pq} \left( {t,v,x} \right)$ determines the probability of the random walker moving from node $v$ to the next node.

- Based on the transition probability $\beta_{pq} \left( {t,v,x} \right)$, start node $t$ and walk step $l$, the random walk sequence of the start node $t$ will be obtained as shown in the Fig. 6.
- Repeat random walk process for each node in the PPI network and obtain several walk sequences for PPI network to represent local structure information.
- Fit the Skip-Gram [46] neural network using the representation vectors of the start node (as input vector) and each node in the random walk sequence of the start node (as output vector).
- Finally, the $k$-dimensional vectors will be used to represent the graph embedding information of the whole PPI network. The feature matrix is $U_{k} \in R^{n \times k}$ as follows:
  6 $$\left[ {\begin{array}{*{20}c} {u_{1,1} } & \cdots & {u_{1,k} } \\ \vdots & \ddots & \vdots \\ {u_{n,1} } & \cdots & {u_{n,k} } \\ \end{array} } \right]$$

### DVA model and cross-validation

We used random forest, a machine learning technique, to predict whether a missense variant is disease-related or neutral. Due to the differences in training samples, tenfold cross-validation for each dataset was performed to enhance the robustness of the results for the DVA method, respectively. The detailed steps of tenfold cross-validation are: (1) randomly divide the entire dataset into 10 groups of equal size; (2) for each unique group: First, take the unique group as the test set. Second, take the remaining groups as the training set. Third, fit the prediction model on the training set and evaluate it on the test set; (3) take the average of ten evaluation results as the final result. We trained random forest on the set of variants by using the *randomForest* [47] package with 500 binary classification trees to predict whether a missense variant is disease-related or neutral. The DVA score represents the fraction of the final prediction score which votes for the variant being disease-related or neutral.

## Data Availability

The datasets for this study can be found in COSMIC (https://cancer.sanger.ac.uk/cosmic), VariSNP (http://structure.bmc.lu.se/VariSNP/index.php) and STRING (https://string-db.org/). The source code of DVA has been uploaded to the GitHub repository (https://github.com/csdwang/DVAscore) and our server (http://bionet.org.cn/DVA).

## Abbreviations

DVA: Disease-related variant annotation
SNV: Single nucleotide variant
ROC: Receiver operating characteristic curve
AUROC: Area under receiver operating characteristic curve
PPI: Protein–protein network
DNN: Deep neural network
AF: Allele frequency
TP: True positive
TN: True negative
FP: False positive
FN: False negative

## References

[1] Davis CA, Hitz BC, Sloan CA, Chan ET, Davidson JM, Gabdank I (2018). The encyclopedia of DNA elements (ENCODE): data portal update. Nucleic Acids Res.

[2] Clarke L, Fairley S, Zheng-Bradley X, Streeter I, Perry E, Lowy E (2017). The international Genome sample resource (IGSR): a worldwide collection of genome variation incorporating the 1000 Genomes Project data. Nucleic Acids Res.

[3] Landrum MJ, Lee JM, Benson M, Brown G, Chao C, Chitipiralla S (2016). ClinVar: public archive of interpretations of clinically relevant variants. Nucleic Acids Res.

[4] Forbes SA, Beare D, Gunasekaran P, Leung K, Bindal N, Boutselakis H (2015). COSMIC: exploring the world’s knowledge of somatic mutations in human cancer. Nucleic Acids Res.

[5] Forbes S, Clements J, Dawson E, Bamford S, Webb T, Dogan A (2006). COSMIC 2005. Br J Cancer.

[6] Kumar P, Henikoff S, Ng PC (2009). Predicting the effects of coding non-synonymous variants on protein function using the SIFT algorithm. Nat Protoc.

[7] Shihab HA, Rogers MF, Gough J, Mort M, Cooper DN, Day INM (2015). An integrative approach to predicting the functional effects of non-coding and coding sequence variation. Bioinformatics.

[8] Ioannidis NM, Rothstein JH, Pejaver V, Middha S, McDonnell SK, Baheti S (2016). REVEL: an ensemble method for predicting the pathogenicity of rare missense variants. Am J Hum Genet.

[9] Chennen K, Weber T, Lornage X, Kress A, Böhm J, Thompson J (2020). MISTIC: a prediction tool to reveal disease-relevant deleterious missense variants. PLoS ONE.

[10] Wang D, Li J, Wang Y, Wang E (2022). A comparison on predicting functional impact of genomic variants. NAR Genom Bioinform.

[11] Roff D (1993). Evolution of life histories: theory and analysis.

[12] Cygler M, Schrag JD, Sussman JL, Harel M, Silman I, Gentry MK (1993). Relationship between sequence conservation and three-dimensional structure in a large family of esterases, lipases, and related proteins. Protein Sci.

[13] Anantharaman V, Aravind L, Koonin EV (2003). Emergence of diverse biochemical activities in evolutionarily conserved structural scaffolds of proteins. Curr Opin Chem Biol.

[14] Alirezaie N, Kernohan KD, Hartley T, Majewski J, Hocking TD (2018). ClinPred: prediction tool to identify disease-relevant nonsynonymous single-nucleotide variants. Am J Hum Genet.

[15] Choi Y, Sims GE, Murphy S, Miller JR, Chan AP (2012). Predicting the functional effect of amino acid substitutions and indels. PLoS ONE.

[16] Schwarz JM, Rödelsperger C, Schuelke M, Seelow D (2010). MutationTaster evaluates disease-causing potential of sequence alterations. Nat Methods.

[17] Reva B, Antipin Y, Sander C (2011). Predicting the functional impact of protein mutations: application to cancer genomics. Nucleic Acids Res.

[18] Quang D, Chen Y, Xie X (2015). DANN: a deep learning approach for annotating the pathogenicity of genetic variants. Bioinformatics.

[19] Dong C, Wei P, Jian X, Gibbs R, Boerwinkle E, Wang K (2015). Comparison and integration of deleteriousness prediction methods for nonsynonymous SNVs in whole exome sequencing studies. Hum Mol Genet.

[20] Rentzsch P, Witten D, Cooper GM, Shendure J, Kircher M (2019). CADD: predicting the deleteriousness of variants throughout the human genome. Nucleic Acids Res.

[21] Sundaram L, Gao H, Padigepati SR, McRae JF, Li Y, Kosmicki JA (2018). Predicting the clinical impact of human mutation with deep neural networks. Nat Genet.

[22] Jagadeesh KA, Wenger AM, Berger MJ, Guturu H, Stenson PD, Cooper DN (2016). M-CAP eliminates a majority of variants of uncertain significance in clinical exomes at high sensitivity. Nat Genet.

[23] Shajani-Yi Z, de Abreu FB, Peterson JD, Tsongalis GJ (2018). Frequency of somatic TP53 mutations in combination with known pathogenic mutations in colon adenocarcinoma, non-small cell lung carcinoma, and gliomas as identified by next-generation sequencing. Neoplasia.

[24] Daboub JAF, Grimmer JF, Frigerio A, Wooderchak-Donahue W, Arnold R, Szymanski J (2020). Parkes Weber syndrome associated with two somatic pathogenic variants in RASA1. Mol Case Stud.

[25] Timms KM, Abkevich V, Hughes E, Neff C, Reid J, Morris B (2014). Association of BRCA1/2 defects with genomic scores predictive of DNA damage repair deficiency among breast cancer subtypes. Breast Cancer Res.

[26] Levy ED, Pereira-Leal JB, Chothia C, Teichmann SA (2006). 3D complex: a structural classification of protein complexes. PLoS Comput Biol.

[27] Burley SK, Berman HM, Kleywegt GJ, Markley JL, Nakamura H, Velankar S (2017). Protein Data Bank (PDB): the single global macromolecular structure archive. Protein Crystallogr.

[28] Jankauskaitė J, Jiménez-García B, Dapkūnas J, Fernández-Recio J, Moal IH (2019). SKEMPI 2.0: an updated benchmark of changes in protein–protein binding energy, kinetics and thermodynamics upon mutation. Bioinformatics.

[29] Nair PS, Vihinen M (2013). VariBench: a benchmark database for variations. Hum Mutat.

[30] Schaafsma GCP, Vihinen M (2015). VariSNP, a benchmark database for variations from db SNP. Hum Mutat.

[31] Adzhubei IA, Schmidt S, Peshkin L, Ramensky VE, Gerasimova A, Bork P (2010). A method and server for predicting damaging missense mutations. Nat Methods.

[32] Li M-X, Kwan JSH, Bao S-Y, Yang W, Ho S-L, Song Y-Q (2013). Predicting Mendelian disease-causing non-synonymous single nucleotide variants in exome sequencing studies. PLoS Genet.

[33] Mottaz A, David FPA, Veuthey A-L, Yip YL (2010). Easy retrieval of single amino-acid polymorphisms and phenotype information using SwissVar. Bioinformatics.

[34] Robin X, Turck N, Hainard A, Tiberti N, Lisacek F, Sanchez J-C (2011). pROC: an open-source package for R and S+ to analyze and compare ROC curves. BMC Bioinform.

[35] Davydov EV, Goode DL, Sirota M, Cooper GM, Sidow A, Batzoglou S (2010). Identifying a high fraction of the human genome to be under selective constraint using GERP++. PLoS Comput Biol.

[36] Hubisz MJ, Pollard KS, Siepel A (2011). PHAST and RPHAST: phylogenetic analysis with space/time models. Brief Bioinform.

[37] Pollard KS, Hubisz MJ, Rosenbloom KR, Siepel A (2010). Detection of nonneutral substitution rates on mammalian phylogenies. Genome Res.

[38] Li Y, Vinckenbosch N, Tian G, Huerta-Sanchez E, Jiang T, Jiang H (2010). Resequencing of 200 human exomes identifies an excess of low-frequency non-synonymous coding variants. Nat Genet.

[39] Karczewski KJ, Francioli LC, Tiao G, Cummings BB, Alföldi J, Wang Q (2020). The mutational constraint spectrum quantified from variation in 141,456 humans. Nature.

[40] You K. filling: matrix completion, imputation, and inpainting methods. 2020.

[41] Wang K, Li M, Hakonarson H (2010). ANNOVAR: functional annotation of genetic variants from high-throughput sequencing data. Nucleic Acids Res.

[42] Liu X, Wu C, Li C, Boerwinkle E (2016). dbNSFP v3.0: a one-stop database of functional predictions and annotations for human nonsynonymous and splice-site SNVs. Hum Mutat.

[43] Liu X, Li C, Mou C, Dong Y, Tu Y (2020). dbNSFP v4: a comprehensive database of transcript-specific functional predictions and annotations for human nonsynonymous and splice-site SNVs. Genome Med.

[44] Grover A, Leskovec J. node2vec: scalable feature learning for networks. In: Proceedings of the 22nd ACM SIGKDD international conference on Knowledge discovery and data mining. 2016. p. 855–64. 10.1145/2939672.2939754 PMC5108654 27853626

[45] Szklarczyk D, Gable AL, Lyon D, Junge A, Wyder S, Huerta-Cepas J (2019). STRING v11: protein–protein association networks with increased coverage, supporting functional discovery in genome-wide experimental datasets. Nucleic Acids Res.

[46] McCormick C. Word2vec tutorial-the skip-gram model. https://mccormickml.com/2016/04/19/word2vec-tutorial-the-skip-gram-model. 2016.

[47] Liaw A, Wiener M (2002). Classification and regression by randomForest. R News.

